# On the cost-effectiveness of insecticide-treated wall liner and indoor residual spraying as additions to insecticide treated bed nets to prevent malaria: findings from cluster randomized trials in Tanzania

**DOI:** 10.1186/s12889-021-11671-2

**Published:** 2021-09-14

**Authors:** Kihomo Robert Mpangala, Yara A. Halasa-Rappel, Mohamed Seif Mohamed, Ruth C. Mnzava, Kaseem J. Mkuza, Peter E. Mangesho, William N. Kisinza, Joseph P. Mugasa, Louisa A. Messenger, George Mtove, Aggrey R. Kihombo, Donald S. Shepard

**Affiliations:** 1 Brandeis University, Waltham, MA 02453 USA; 2 Empowered and Improvement Livelihood Initiatives Foundation, Dar es Salaam, Tanzania; 3 National Institute for Medical Research, Amani Centre, Muheza, Tanzania; 4 Population Services International, Dar es Salaam, Tanzania; 5 London School of Tropical Medicine and Hygiene, London, UK; 6 Mzumbe University, Dar es Salaam, Tanzania

**Keywords:** Malaria prevention, Cost, Tanzania, Insecticide-Treated Wall liner, Indoor residual spraying, Vector control, Cost-effectiveness, Randomized controlled trial, RCT, Pyrethroid resistance

## Abstract

**Background:**

Despite widespread use of long-lasting insecticidal nets (LLINs) and other tools, malaria caused 409,000 deaths worldwide in 2019. While indoor residual spraying (IRS) is an effective supplement, IRS is moderately expensive and logistically challenging. In endemic areas, IRS requires yearly application just before the main rainy season and potential interim reapplications. A new technology, insecticide-treated wall liner (ITWL), might overcome these challenges.

**Methods:**

We conducted a 44-cluster two-arm randomized controlled trial in Muheza, Tanzania from 2015 to 2016 to evaluate the cost and efficacy of a non-pyrethroid ITWL to supplement LLINs, analyzing operational changes over three installation phases. The estimated efficacy (with 95% confidence intervals) of IRS as a supplement to LLINs came mainly from a published randomized trial in Muleba, Tanzania. We obtained financial costs of IRS from published reports and conducted a household survey of a similar IRS program near Muleba to determine household costs. The costs of ITWL were amortized over its 4-year expected lifetime and converted to 2019 US dollars using Tanzania’s GDP deflator and market exchange rates.

**Results:**

Operational improvements from phases 1 to 3 raised ITWL coverage from 35.1 to 67.1% of initially targeted households while reducing economic cost from $34.18 to $30.56 per person covered. However, 90 days after installing ITWL in 5666 households, the randomized trial was terminated prematurely because cone bioassay tests showed that ITWL no longer killed mosquitoes and therefore could not prevent malaria. The ITWL cost $10.11 per person per year compared to $5.69 for IRS. With an efficacy of 57% (3–81%), IRS averted 1162 (61–1651) disability-adjusted life years (DALYs) per 100,000 population yearly. Its incremental cost-effectiveness ratio (ICER) per DALY averted was $490 (45% of Tanzania’s per capita gross national income).

**Conclusions:**

These findings provide design specifications for future ITWL development and implementation. It would need to be efficacious and more effective and/or less costly than IRS, so more persons could be protected with a given budget. The durability of a previous ITWL, progress in non-pyrethroid tools, economies of scale and competition (as occurred with LLINs), strengthened community engagement, and more efficient installation and management procedures all offer promise of achieving these goals. Therefore, ITWLs merit ongoing study.

**First posted:**

2015 (NCT02533336).

**Supplementary Information:**

The online version contains supplementary material available at 10.1186/s12889-021-11671-2.

## Background

Vector control is an essential component of the global strategy for malaria control aiming to avert parasite transmission through interventions targeting adult anopheline vectors [1]. Two vector control approaches, long-lasting insecticide treated nets (LLINs) and indoor residual spraying (IRS), have substantially reduced malaria morbidity and mortality since 1990 [2, 3]. Nevertheless, this disease remains a major health challenge, especially in sub-Saharan Africa [3, 4]. The disease still caused 409,000 deaths globally in 2019 [5]. Increasing mosquito insecticide resistance, especially to pyrethroid products, threatens the long-term effectiveness of both LLINs and IRS [3, 6–8]. IRS is moderately expensive and logistically challenging. As IRS provides only a few months of protection [9], it must be applied just before the rainy season(s) and potentially requires multiple reapplications per year in endemic areas.

To address these challenges, researchers developed a new technology: the non-pyrethroid insecticide-treated wall liner (ITWL). Early research showed that local nails with a plastic nail cap could affix ITWL to the interior of a mud wall [3]. Subsequent research showed that the product was well accepted by users and could serve as a complement to LLINs [3, 10–12]. An ITWL offers several logistical advantages over IRS. The ITWL’s expected multi-year efficacy avoids the complexity of repeated rounds of application in the same and successive years [7]. Also, ITWL installation is not time sensitive. As a complement to LLINs, ITWLs were expected to protect household members before going to bed, those not using any LLIN or utilizing a damaged LLIN, and addressed mounting insecticide resistance to pyrethroids [3, 7]. We are aware of only a single epidemiological efficacy study of ITWLs in Africa as a supplement to LLINs [13]. That cluster randomized trial randomized 12 paired clusters with 1592 children between intervention (ITWL plus LLIN) and control (LLINs only) conditions. Conducted near Asembo (Nyanza Province), Kenya before the onset of pyrethroid resistance, that trial found that the pyrethroid ITWL had a 38% overall protective efficacy, with 31% efficacy among children under 5 years and 42% among those aged 5–11 years [13]. These findings revealed that the pyrethroid ITWL technology had promise and was likely to be cost-effective in this site without resistance [12]. An evaluation of the same product from Balaghat, India, subsequently published, gave comparable results [14].

As described below, in the face of pyrethroid resistance, a non-pyrethroid ITWL was developed and deployed in a 44-cluster randomized trial in Muheza District, Tanzania, but found to be ineffective [7]. In spite of this deficiency, the success of a previous ITWL and continuing insecticide development suggests that a future ITWL could potentially be effective against malaria and other vector borne diseases [5, 10]. However, the usefulness of any malaria control tool also depends on its cost and comparison with alternative approaches. As a similar evaluation of the cost and effectiveness of a new technology would likely take years and cost millions of dollars to plan, implement and evaluate, it is critical to document all past initiatives, including failures, to advance malaria control. Thus, this paper first reports the economic cost of installing (with improvements over three phases) and removing ITWL in the Muheza trial [7]. Next, it uses the lessons learned to project the cost of a future efficient installation. Additionally, it examines the cost and effectiveness of IRS from Muleba, Tanzania, another district with endemic malaria. Finally, using IRS as a benchmark, this paper assesses the cost and cost-effectiveness of alternative product profiles of future potential ITWL installations.

## Methods

### Design of cluster randomized trial of ITWL

The cluster randomized trial of ITWL was planned for Muheza district. Muheza is located 35 km west of Tanga City, the capital of Tanga Region and 364 km north of Dar es Salaam. It had a 2012 population of 204,461 residents (100,843 males and 103,618 females). With an area of 1498 km^2^, its density was 136 inhabitants per square kilometer [15].

Malaria is endemic in the district, with the main vectors being *An. gambiae s.l.* and *An. funestus s.l*. The trial initially planned to use the pyrethroid product that had proved efficacious in Kenya [13]. During the planning phase of the Muheza trial, however, entomological data showed that pyrethroid resistance had been documented, indicating that a pyrethroid product might not be effective [16]. In response, implementation was delayed while the ITWL supplier, Vestergaard (formerly Vestergaard Frandsen) in Switzerland, developed a new ITWL designed to address insect resistance to pyrethroids.

The final ITWL product was composed of a high-density polypropylene non-woven fabric containing a proprietary combination of two non-pyrethroid insecticides (0.25% abamectin and 1% fenpyroximate) [17]. The side of this fabric that is attached to inside house walls is inactive, moisture resistant, dust free, and thermo-stable. The side which faces the house interior is the active one, functioning as a long-lasting insecticidal reservoir containing the insecticidal mixture embedded in a polymer. A pilot study found that a prototype of this product (termed PermaNet Lining) was well accepted by rural African households [3, 9].

The protocol was updated to ensure statistical power as a two-arm 44-cluster randomized trial with 22 intervention and 22 control clusters comparing the existing standard of care (LLINs) against the experimental intervention (ITWL plus LLINs). To ensure the accuracy of this comparison, in August 2015 all enumerated households in both experimental and control arms were given one Interceptor® LLIN (BASF Corporation, Germany) for every two persons and instructed on their use. These nets contained alphacypermethrin (200 mg/m^2^) coated onto polyester fibers. The primary planned endpoint was the cumulative one-year incidence of parasitemia in children aged 6–59 months assessed through a malaria rapid diagnostic test administered at monthly household visits. The trial, NCT02533336, was first posted on 26/08/2015 and the protocol was published [7]. Supplemental Figure S1 shows the trial’s CONSORT diagram.

### Implementation of ITWL

Implementation in experimental clusters began with recruitment of 140 installers. Next, professionals from the sponsoring organization, the National Institute for Medical Research (NIMR) and a consultant who had managed the previous Asembo trial conducted a 5-day training session for the installers. The installers needed to be residents of the study villages and most were males. The installers needed to be capable of manual labor and have a basic knowledge of carpentry, but had no specific educational requirement. The training provided an overview of the project, and instruction and practice on the installation of ITWL. This entailed measuring the rooms; cutting the ITWL (which came on rolls about 2 m wide); identifying standard intervals for nails; attaching the material to walls while avoiding damage to the houses, household items, and the environment; and documenting the work. As manpower needs grew, some of the original installers were promoted to team leaders or supervisors and additional installers were added and trained on the job. Supervisors needed to be literate so they could complete the necessary forms.

Supervisors visited households in experimental clusters ahead of the planned installation exercise to describe the process and request consent from the household head. They assigned the installation teams to specific houses that consented to have ITWL installed, monitored the installation process, and approved installers’ payment on confirmation of the completion of work. The team leaders guided teams, measured the walls, windows and doors of each house, and ensured timely completion of each day’s work. Throughout the installation, five full-time NIMR staff oversaw the work, covering epidemiology, entomology, sensitization, installation, logistics, and finances.

### Updating to current epidemiologic and economic conditions

To facilitate the interpretation of this study, we have adjusted all epidemiologic and economic information to values for 2019, the most recent year with comprehensive data, expressing costs in current 2019 US dollars (USD). We converted monetary amounts to 2019 USD through four steps: (1) We converted primarily domestic costs reported in USD to the equivalent in Tanzanian shillings (TZS) in the same year using the applicable exchange rate. The applicable exchange rate (TZS/USD) for the organization installing the wall liner, NIMR, was the net rate received by its bank (i.e., 2074.1 in 2015 and 2065.0 in 2016) while the applicable rate for other sources was the official 2011 rate (1572.1) [18]. (2) We converted TZS in any year prior to 2019 to 2019 TZS based on the Tanzanian official gross domestic product (GDP) deflator in that year and in 2019 (i.e. 72.333 in 2011, 100.000 in 2015, 107.472 in 2016, 121.006 in 2019) [19]. (3) We converted 2019 TZS to 2019 USD based on the 2019 exchange rate at 1.00 USD equals 2288.1 TZS [20]. (4) We converted prices of primarily international inputs to 2019 USD based on the US GDP deflator [19]. For comparative indicators, we used Tanzania’s 2019 per capita gross national income (GNI) ($1080) [21]. We adjusted epidemiologic information based on malaria’s 2019 burden of 2038 disability-adjusted life years (DALYS) per 100,000 population per year [22].

### ITWL intervention phases

Installation of ITWL in experimental clusters was conducted in three phases totaling 204 days, characterized by distinct modes of sensitization and payment. The first installation phase, August 10, 2015 through December 4, 2015 (117 days), used large community meetings to try to sensitize residents about the desirability of ITWL. Village leaders, community health workers (CHWs), and researchers used these meetings to try to inform residents about the product and installation schedule. These meetings also sought to raise awareness, enhance participation in the trial, and increase the use of bed nets. This phase involved 352 workers (220 installers, 110 team leaders, and 22 supervisors) each paid 10,000 TZS per day. ($4.82 in 2015, equivalent to $5.29 in 2019 USD). However, NIMR staff observed that the daily wage system appeared to create a perverse incentive for installers to work slowly so as to maximize their number of work days.

The second installation phase, December 5, 2015 through January 15, 2016 (42 days), aimed at raising the number of community members participating in the project. This phase introduced better personal protective equipment (flexible gloves) for installers, added a megaphone so that project staff could better attract residents’ attention, and initiated door-to-door visits and sensitization to explain the product and answer questions in detail. It also sought to improve the efficiency of the installation. This phase had only 242 workers (22 supervisors and 220 installers). Also, the payment system was changed from a daily wage to an output-based payment, with each supervisor receiving 1500 TZS ($0.72 in 2015 USD, equivalent to $0.79 in 2019 USD) and each team of installers receiving 7000 TZS ($3.37 in 2015, equivalent to $3.70 in 2019 USD) for each installed house.

The third installation phase, January 16, 2016 through February 29, 2016 (45 days), involved additional sensitization approaches. It added the distribution of brochures with photographs and simply-worded Swahili explanations of ITWL benefits. Members of the project’s socio-economic team continued to make announcements throughout the village with a portable megaphone to increase residents’ willingness to have the product installed in their homes. While maintaining the previous phase’s piecework payment modality, the third phase sought to reduce costs per household further by lowering the number of personnel to 154 workers (14 supervisors and 140 installers).

### Entomologic results and de-installation

At 2 months after installation, an entomological trial in experimental huts in Zeneti, near Muheza, of the incremental benefit of alternative ITWL products over LLINs alone found no benefit of the pyrethroid product due to insecticide resistance but a small, though not statistically significant incremental benefit of the non-pyrethroid product on mosquito mortality [23]. Noting that results at 2 months were not necessarily predictive of longer term results, the investigators initiated the cluster randomized epidemiologic trial in Muheza with this non-pyrethroid product in 2015.

However, the entomological results from this trial that emerged in May 2016 showed that this wall liner was no longer effective. Cone bioassay tests at 90 days after installation found that ITWL no longer killed mosquitoes in residents’ houses in Muheza district, perhaps due to issues with degradation of chemical content and/or bioavailability of the insecticides in the ITWL, such that mosquitoes did not obtain a lethal dose upon contact. Entomological studies on mosquito age confirmed the lack of efficacy [16].

As a result, the study’s data safety monitoring board, investigators and sponsors determined that the study needed to be terminated prematurely. Collection of epidemiologic data was stopped. Since ITWL was no longer beneficial and potentially harmful, they concluded later in 2016 that the ITWL should be removed from residents’ houses where possible [8].

De-installation of ITWL material lasted from September 21, 2016 through October 6, 2016 (16 days). It involved 13 regular NIMR staff. The de-installation phase began by a three-day training by NIMR staff of 220 de-installers and 22 cluster supervisors. Next, community residents were invited to sensitization sessions, aided by project staff, community leaders and CHWs, to explain why ITWL was being removed prematurely.

### Determining economic costs of the ITWL intervention

For analytic purposes, the unit of analysis was a household, defined as a group of people who live together and share food and expenses. In small villages, each household generally owned its own house (a building). However, in large villages and small towns, multiple households could share a house. In most cases, the household members were related to one another, so they also constituted a family. The sample for the intervention arm consisted of the 5666 households in experimental clusters who had the ITWL installed in their house. These installed households represented 67.1% of the 8444 initially targeted households in experimental clusters, and 76.8% of the 7373 households finally enumerated in those clusters. The remaining households had moved, could not be reached, or did not consent to installation.

The NIMR office at Muheza provided aggregate financial expenditures for all project activities serving these households. Non-financial data, on the other hand, represented the opportunity cost of contributed labor used in the intervention. Obtaining this information required interviewing respondents from a sample of households using this technology, and similar questions for households using other technologies being examined. As the authors were not testing a specific hypothesis, the necessary sample size could not be calculated with a conventional power analysis. Instead, the authors selected the largest sample possible with available study resources, establishing similar sample sizes for ITWL and other technologies. Accordingly, research staff from the project’s socio-economic team interviewed 136 households using questionnaires and recorded their observations. The authors obtained qualitative data through regular interactions with residents and community leaders.

The questionnaire assessed the non-financial costs in terms of the average time spent and the number of household members involved in the following activities: (i) attending sensitization meetings, (ii) consenting, (iii) removing household items before the installation took place, (iv) waiting for the installers to arrive and complete their work, (v) putting household items back after ITWL installation, (vi) removing household items before de-installation, (vii) waiting for the de-installers to arrive and/or assisting them or waiting for them to complete their work, and (viii) putting household items back after ITWL de-installation.

We then imputed and valued the aggregate and average time for the 5666 households. The number of households in which ITWL was installed was derived from records of payments to installers. While Vestergaard donated the ITWL products for this trial, we imputed its cost based on a related product. Vestergaard had previously marketed the pyrethroid ITWL used in the Asembo study under the name ZeroVector [14]. As both products had comparable purposes, settings (rural areas with mostly mud houses), and methods of supply (rolls about 2 m wide and 100 m long), we used the average product cost per household in Kenya based on that product’s latest sales price per roll ($68.50) as the estimate for this study [8].

To compute the cost of time households spent during sensitization, installation, and de-installation exercises, we used Tanzania’s 2015 daily wage rate of TZS 6581 for a typical laborer from nearby sisal estates in Muheza district, obtained as part of the interviews in this study. Since none of the resources used in installing and de-installing ITWL involved capital inputs, we considered all costs as occurring at the time of installation. We added 15% of the direct expenditure(s) for overhead based on the rate allowed by the main sponsor of this economic assessment for expenses in Tanzania [24]. These overhead costs covered utilities, facilities upkeep, and central administration.

### Efficacy and cost of IRS in Tanzania

Information on the efficacy and financial costs of the comparative intervention (IRS) came from existing publications. Tanzania had previously hosted a cluster randomized trial of the incremental efficacy of IRS as an addition to LLINs. That study was conducted near Muleba, Tanzania (in the country’s Lake Region). The intervention arm, entailing rounds of spraying conducted prior to the long and short rains, followed the same two-round schedule as another intervention study in the same district [25]. In the randomized trial, IRS plus LLINs reduced malaria incidence by 57% compared to the control arm with only LLINs, a significant change, with a 95% confidence interval of 3–81% [26]. We multiplied the best estimate of efficacy times the DALY burden in Tanzania to get the best estimate of the DALYs averted and used the confidence interval on efficacy to generate the confidence interval on DALYs averted.

To estimate the financial cost of IRS, we extracted information from a modeling study from 2008 to 2012 of the combination of IRS plus LLIN and of LLIN alone in mainland Tanzania (including the vicinity of Muleba), reported in 2011 US dollars [27]. We used the version of their analysis that included the cost of the more expensive insecticides to address insect resistance to pyrethroids. Their analysis generally followed the framework of integrated vector management and incorporated the value of in-kind contributions from government personnel and community leaders [28]. However, their analysis did not include a non-financial component, the economic value of household time for IRS.

As an additional component of the ITWL economic evaluation, we designed and implemented a survey of households who had received IRS. After obtaining permission from the government and local leaders, we used a cluster sample to select 3 districts in the vicinity of Lake Victoria, Tanzania. From these districts, we randomly selected 29 wards, then randomly chose 5 households from each ward, and invited the chosen households to participate in this costing survey. From the 145 households invited to participate, 135 households (93%) completed the IRS survey. This final sample size was just 1% below that of the ITWL household survey. A research team member interviewed a member of each participating IRS household in person between December 2015 and January 2016. The survey collected information about the time household members spent attending informational meetings with government officials regarding IRS, providing 20 l of water per household (needed by spray operators to mix with the insecticide), removing and replacing furniture, awaiting the spraying operator, being present during spraying, waiting for 2 hours or more after spraying (with windows and doors open), and cleaning up dead insects. We valued their time at the 2015 hourly minimum wage of TZS513 ($0.2713 in 2019 USD).

### Cost-effectiveness framework for future ITWL products

As data on costs of ITWL in Muheza were collected in the context of a randomized trial focusing on evaluating the efficacy of a new product, the implementers did not yet have the context or experience on how best to implement the intervention most efficiently or at scale. Therefore, installation costs per household in the trial were likely higher than those expected after the managing organization gained more experience. Similarly, a previous study found that researchers can use lessons learned from a trial to plan the delivery of the key products or services in an “adapted community model.” [29] Adapted community models thus show a tendency to be comparably effective but less costly per participant. To extend our findings beyond the trial, we examined the components of costs to identify potential efficiencies for use of a future ITWL product.

We expressed incremental cost-effectiveness ratios (ICERs) per DALY averted both in US dollars and as multiples of Tanzania’s 2019 per capita gross national income (GNI, $1080).

### Ethical approval

The study was approved by the ethics committees of the NIMR, Kilimanjaro Medical College, the London School of Tropical Medicine and Hygiene, and the Brandeis University Human Research Protection Program.

## Results

### ITWL coverage

**Fig. 1:**
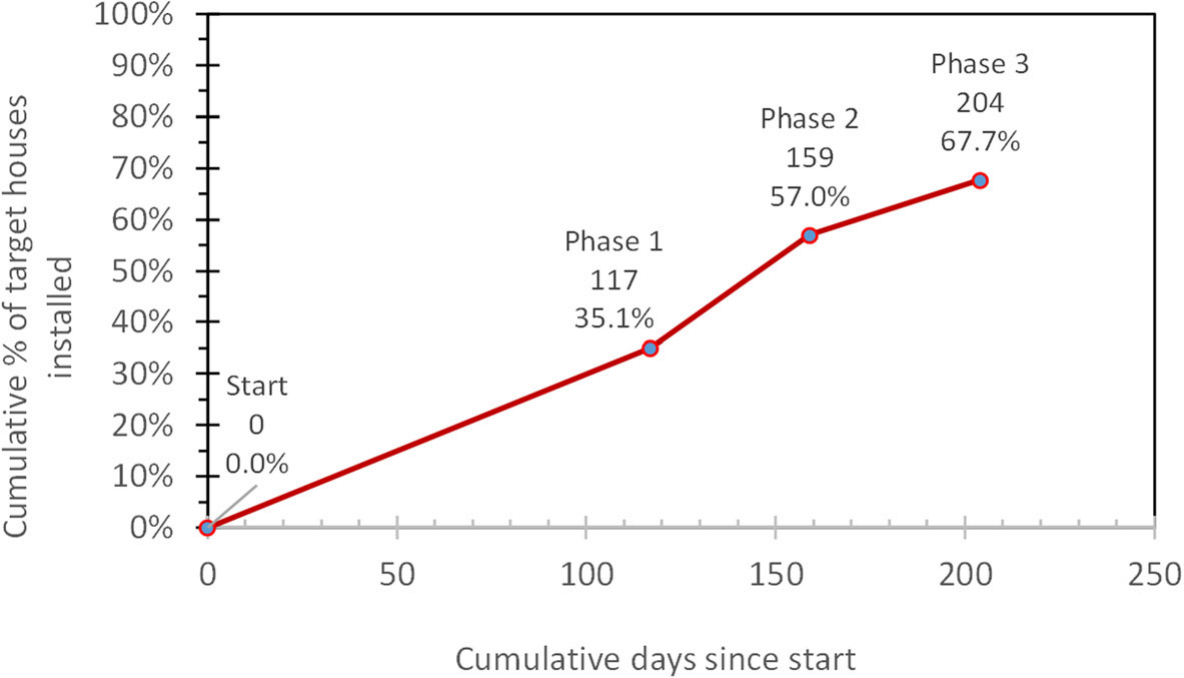
Progress on wall liner installation by phase (labels show cumulative numbers of days since start and cumulative percentage of target households installed)

The phasing of installation stages provided the opportunity to compare the increasingly stronger approaches to community sensitization and more efficient modalities of payment to installers. Initially, 8368 households in the district were targeted to receive ITWL, an average of 279 per intervention cluster. In the end, the study completed installation in 5666 households, 67.7% of the initial target. Figure 1 shows the cumulative coverage across the three installation phases, each phase adding a notable increase in coverage, thanks to better management, enhanced sensitization, and incentives to installers.

In the first phase, many households refused installation because they observed that some of the installers experienced rashes on their arms, hands, and sometimes their private parts. This occurred because they had not been given adequate personal protection equipment (gloves and long-sleeved shirts) and sufficient instruction about removing gloves prior to relieving themselves. These symptoms created fears and initiated rumors that ITWL caused male sterility. Attendance at the community meetings was low, as Tanzania was then in midst of a high-profile presidential election campaign. As a result, phase 1 attained only 35.1% coverage. The door-to-door sensitization and other additions in the second phase rapidly raised coverage to 57.0%. The brochures and other refinements in phase 3 increased coverage further to 67.7% of eligible households. The main barriers in the remaining households were nicely finished walls (where the owners did not want the walls disfigured by nails or covered by ITWL), or occupancy by tenants (who lacked the right to approve installation of ITWL).

### ITWL installation costs

In the first phase, during which installers were paid by a daily wage, 2933 households were installed in 117 days, or 25.1 households per day. This phase resulted in 0.071 households installed per worker per day at an economic cost of $136.72 per household in 2019 prices (see installation phases columns in Table 1). In the second phase, workers installed 1835 households in 42 days, or 43.7 households installed per day. The rate was 0.181 households installed per worker per day at an economic cost of $135.01 per household. In the third phase, workers installed 898 houses in 45 days, averaging 20.0 households per day or 0.130 households per worker per day at an economic cost of $122.25 per household installed. As the most willing houses were installed over successive clusters in phases 1, 2, and 3 and the most reluctant houses were installed last, installation likely faced the greatest challenges in phase 3. Nevertheless, improved management and piecework payment increased the pace of installation and productivity per worker from phase 1 to phase 2. The extra effort in persuading reluctant households and schedule uncertainty likely lowered the pace and productivity from phase 2 to phase 3. However, under the output payment system, personnel and local transport cost per installed house fell compared to phase 2. In summary, the economic cost per house averaged $136.38 over the installation portion of ITWL, consisting of financial costs of $132.19 (97%) and non-financial costs of $4.19 (3%).

**Table 1:** Installation and de-installation costs in 2019 US$ based on installed households by phase and input

Input	Installation phases				De-installation phases				Combined	
	Phase 1	Phase 2	Phase 3	Average	Sensi-tization	Pilot	Full scale	Sum	Cost	Col. %
Cost per household covered										
Personnel	$35.86	$28.21	$24.72	$31.61	$0.61	$0.21	$5.96	$6.79	$38.40	24.4%
Materials	$13.61	$13.61	$13.61	$13.61	$0.00	$0.31	$2.28	$2.59	$16.20	10.3%
Local transport	$3.49	$10.23	$1.66	$5.37	$0.31	$0.00	$4.21	$4.52	$9.89	6.3%
Transfer from port	$4.07	$4.07	$4.07	$4.07	$0.00	$0.00	$0.00	$2.33	$6.40	4.1%
Training	$1.43	$1.43	$1.43	$1.43	$0.00	$0.00	$0.00	$0.00	$1.43	0.9%
Communications	$0.97	$0.97	$0.97	$0.97	$0.00	$0.00	$0.00	$0.00	$0.97	0.6%
Incineration	$0.00	$0.00	$0.00	$0.00	$0.00	$0.00	$2.33	$2.33	$2.33	1.5%
**In-country**	**$59.42**	**$58.52**	**$46.45**	**$57.06**	**$0.92**	**$0.53**	**$14.78**	**$18.56**	**$75.62**	**48.1%**
Wall liner	$75.13	$75.13	$75.13	$75.13	$0.00	$0.00	$0.00	$0.00	$75.13	47.8%
**Subtotal (financial)**	$134.55	$133.65	$121.58	$132.19	$0.92	$0.53	$14.78	$18.56	$150.75	96.0%
Household time	$2.17	$1.36	$0.67	$4.19	$0.98	$0.00	$1.17	$2.15	$6.34	4.0%
**TOTAL (economic)**	**$136.72**	**$135.01**	**$122.25**	**$136.38**	**$1.90**	**$0.53**	**$15.94**	**$20.71**	**$157.09**	**100.0%**
Cost per person covered										
**Subtotal (financial)**	$33.64	$33.41	$30.40	$33.05	$0.23	$0.13	$3.69	$4.64	$37.69	96.0%
**Household time**	$0.54	$0.34	$0.17	$1.05	$0.25	$0.00	$0.29	$0.54	$1.58	4.0%
**TOTAL (economic)**	$34.18	$33.75	$30.56	$34.10	$0.48	$0.13	$3.99	$5.18	$39.27	**100.0%**
Row %	**87.0%**	**85.9%**	**77.8%**	**86.8%**	**1.2%**	**0.3%**	**10.1%**	**13.2%**	**100.0%**	

The 2015 FOB price for the Zero Vector was US$274 per roll (100 m × 2.3 m). All calculations are based on the 5666 installed households

### ITWL de-installation costs

The de-installation phase initially targeted all 5666 installed households with wall liners installed. However, at the de-installation phase, 165 houses were gone (burned, demolished, or relocated) and 153 had unknown status (locked or information not reported). The ITWLs in the remaining 5348 households with data were removed by paid installers (90.9%), removed by household members themselves (8.8%), or retained on the walls at the household’s request and contrary to program recommendations (0.4%). The de-installation phases (columns of Table 1) show that the economic cost per installed household for de-installing ITWL totaled $20.71, with financial costs of $18.56 (89.6%) and non-financial costs of $2.15 (10.4%).

The combined economic costs per house of installing and de-installing the wall liner were $157.09 per household (see combined columns of Table 1). It comprised installation cost of $136.38 (86.8%) and de-installation costs of $20.71 (13.2%). Due to its need for materials and more labor, ITWL installation had an economic cost per house that was almost 7 times that of de-installation.

Of total economic costs for installation and de-installation per household combined, the greatest portion of costs was the imputed cost of ITWL material (47.8% of the total cost), followed by personnel (24.4%), and other materials and supplies (10.3%). Local transport (6.3%), transfer from the port (4.1%), training (0.9%), communications (0.6%), and incineration (1.5%) comprised the remaining 13.4% of the total costs. Field costs, excluding the cost of ITWL itself, were $75.62 per household. The cost per person covered (bottom panel of Table 1) is based on an average of four family members in a typical household in Muheza district [15]. The per-person financial and non-financial costs for installing ITWL were $34.10, while those for de-installing the material were $5.18, summing to a combined cost of $39.27 ($37.69 financial and $1.58 household time) per person.

The analysis of installation by project phases provides insights about project management and promising implications for a future adapted model, as shown in Table 2. The change in mode of payment from days worked to households installed and a reduction in the number of installers in the second phase were implemented abruptly. This resulted in some installers going on strike, disrupting the project’s timetable. A future project may wish to establish and explain a payment plan based on houses installed and not reneging on previous promises. As payment based on the number of households installed creates an incentive to cut corners, regular supervision would be essential.

**Table 2:** Summary of installation phases and implications for an adapted model

Item	Phase 1	Phase 2	Phase 3	Adapted Model
Dates	10 Aug 2015–4 Dec 2015	5 Dec 2015–15 Jan 2016	16 Jan 2016–29 Feb 2016	Future
Sensitization	Community meetings	Door-to-door	Door-to-door & distribution of brochures	Community meetings, megaphone, door-to-door, radio, posters, involvement of community leaders, NGOs, CBOs, FBOs, political and religious leaders, and district and regional officials
Installers’ protective equipment	Non-breathable nylon gloves & inadequate supplies of overalls, safety glasses, & masks.		Adequate supplies of protective equipment	Adequate overalls, safety glasses, masks, & flexible breathable nylon gloves
Management	Transportation delays, late payments to installers, unclear contracts, procurement shortfalls & inadequate supervision		Delays in payments to installers	Clear contracts, timely payments to installers, efficient procurement, carefully supervised staff & organizations, including sub-contracting some activities to experienced organizations

*NGO* denotes non-governmental organization, *CBO* denotes community-based organization, *FBO* denotes faith-based organization

The breakdown by phase also highlights the impact of improvements in logistics and personal protective equipment over successive installation phases. During the first two phases of this project, some installers lacked overalls, eye protection, masks, and breathable nylon gloves, resulting in slowed work and skin rashes. Insufficient planning of transportation also caused delays, as staff in disparate locations had to share one vehicle. This situation resulted in some installers waiting at the office for several hours before the scheduled vehicle returned. Late arrivals of installers also kept household members waiting up to 4 hours for their arrival. Better information, equipment, planning, and management in subsequent phases gradually mitigated these problems.

### Cost-effectiveness of IRS

The financial cost per person In 2011 prices of the combination of IRS and LLIN was $7.49 while that of LLIN alone was $3.41 [27], indicating the net cost of IRS to the public health system of $4.08 (equivalent to $4.65 in 2019 prices). Our household survey found that each household spent an average of 7.67 person-hours per round of IRS moving belongings in and out of the area to be sprayed and procuring the required 20 l of water per household. With four persons per household and two rounds per year, the financial burden on households was 3.835 h per household member per year and $1.04 per person. The total economic cost of IRS was $5.69 per person per year in 2019 prices. The insecticides used were consistent with recommendations of the World Health Organization [30]. Table 3 details the annual cost of ITWL using national data for Tanzania in the trial for IRS.

**Table 3:** Annual cost of insecticide-treated wall liner (ITWL) and indoor residual spraying (IRS) in Tanzania (2019 USD)^a^

Item	ITWL	IRS
Initial economic cost per person (2019 USD)	$39.27	$5.69
Years of protection for one round	4.00	1.00
Annualizing factor at 3% annual discount rate	0.2612	1.000
Cost per person per year (2019 USD)	$10.26	$5.69
Cost relative to ITWL	100.0%	55.4%

^a^ Annualizing calculations assume that payments are made at the beginning of each year. USD denotes United States dollars

The cost of IRS was 55.4% that of ITWL (i.e., $5.69 /$10.26). The best estimate of the health impact of IRS was 1162 DALYs averted per 100,000 population but with wide lower and upper uncertainty estimates of 611 and 1886, respectively [26]. The best estimate of the ICER was $490 (i.e., $5.69 / 1162 × 100,000) with a broad uncertainty range of $344 to $9301. As a multiple of Tanzania’s per capita 2019 GNI ($1080), the best estimate was 0.45 with an uncertainty range of 0.32 to 8.61. The World Health Organization’s Commission on Macroeconomics in Health recommended that if an intervention’s ICER was below 1.00 times the country’s per capita GDP, the intervention should be considered highly cost-effective and recommended [31]. While some researchers acknowledged that other factors also need be considered, many studies used this threshold in cost-effectiveness publications [32]. Actual investment decisions in low- and middle-income countries revealed an actual threshold of 60–65% of GDP per capita [33]. Under the best estimate, as this multiple is below 1.00, IRS as conducted in this Tanzanian program was highly cost-effective according to both the Commission criterion and consistency with actual investment decisions.

### Implications for the target product profile of a future ITWL

**Fig. 2:**
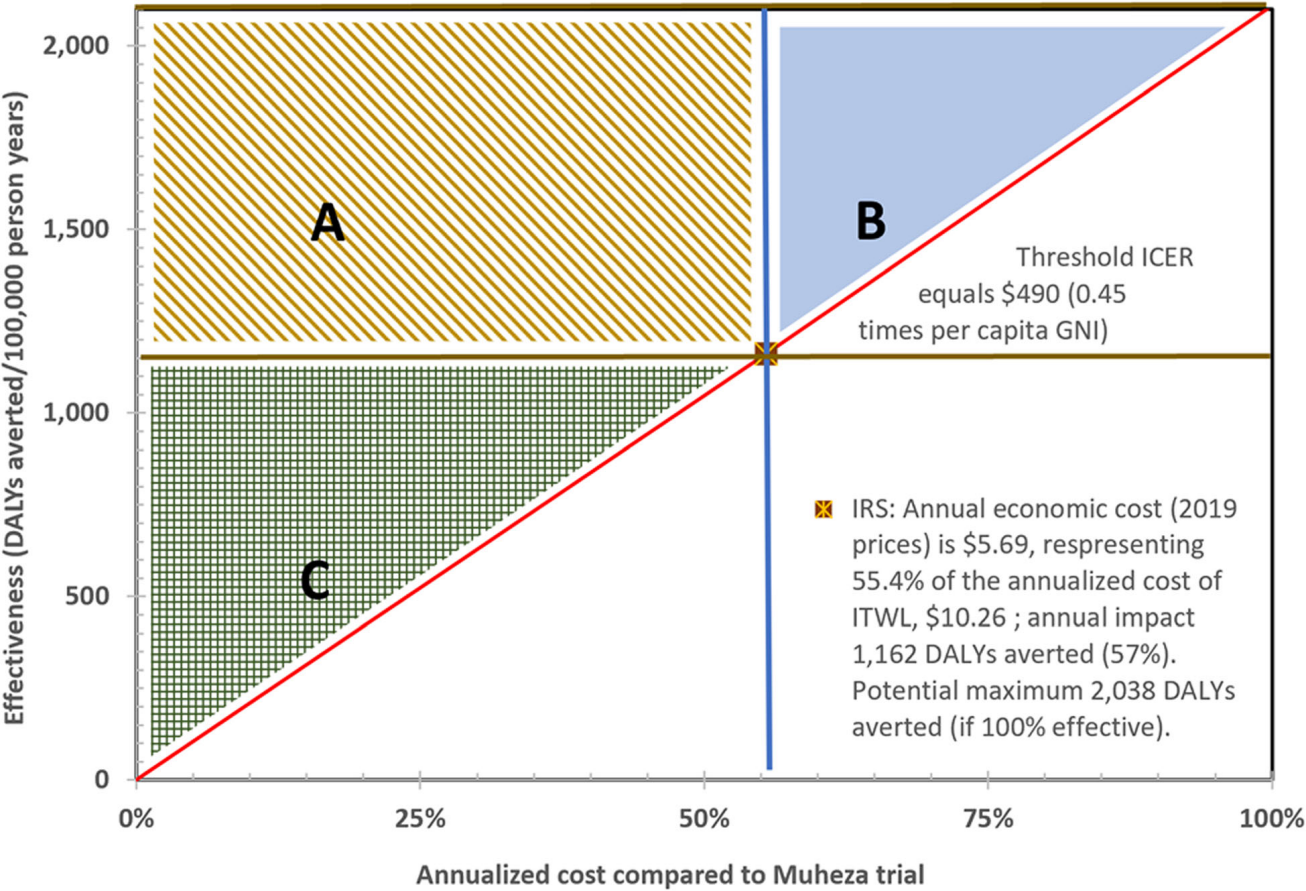
Situations (zones A and C, shown by shading) in which an insecticide treated wall liner (ITWL) would be more cost-effective than and preferable to IRS

As this analysis suggests that IRS was both an effective and cost-effective technology for vector control, IRS serves as a standard for evaluating alternative product profiles of a future ITWL product. Figure 2 provides a framework for this assessment. The key dimensions are the annualized cost, shown on the X-axis relative to the cost of ITWL (range 0–100%) and the annual effectiveness, shown on the Y-axis, ranging from 0 to 2038 DALYS per 100,000 population, corresponding to 0 to 100% of the effectiveness of IRS. The diagonal line, with the slope of $490 per DALY, corresponds to the best estimate of the ICER of IRS. This framework creates three zones, all above this diagonal line, in which a future ITWL product would be more cost-effective and preferable to IRS, designated by the three shaded blocks.

Rectangular zone A represents situations of pure dominance by an ITWL, where a future ITWL product is both more effective and less costly than IRS. In this case, ITWL would be unequivocally preferable to IRS. Triangular zone B represents situations in which a future ITWL is more costly than IRS, but sufficiently more effective to more than justify the added cost. Triangular zone C depicts scenarios in which a future ITWL saves money compared to IRS. While ITWL is less effective than IRS, the cost savings are sufficient to more than justify the sacrifice in effectiveness. In all three shaded zones, if a country had a limited budget for vector control as a complement to LLINs against malaria, a future ITWL would be preferable to current IRS. Conversely, for all combinations below the diagonal line, shown unshaded, ITWL would be less cost effective than IRS and not recommended.

## Discussion

The 38% efficacy of the earlier, pre-resistance version of ITWL in Asembo falls within the wide 95% confidence interval for the effectiveness of IRS in Muleba (3 to 81%) [26]. Also, the Asembo ITWL remained installed, accepted, and in good condition in a number of the houses 5 to 7 years after installation, according to reports from site visits in 2017 (Maurice Ombok, Kenya Medical Research Institute, personal communication, October, 2017). These findings and advances in non-pyrethroid insecticides [30] suggest that a future ITWL has the potential for comparable or superior effectiveness compared to IRS. However, when projected over its 4-year expected life, the cost per person per year of ITWL in Muheza ($10.26) would need to be reduced by 44.6% to match that of IRS ($5.69). To guide future planning, it is useful to examine whether and how a future ITWL could meet the dual requirements of an effective and cost-effective product.

As non-pyrethroid insecticides tend to be more expensive than pyrethroid ones, a new ITWL product might initially be costlier than that in Asembo. However, promising efficiencies could lower the installed cost of a future ITWL. The ITWL installed in Muheza was a new product and its installation in 5666 households was NIMR’s largest such activity to date. The 10.6% decline in cost per house from phase 1 to phase 3 suggests that just a few months of experience can generate notable savings in installation costs. The sharp fall in price of a related product, the LLIN, indicates how the price of the ITWL product itself could be reduced. The price per net purchased by a major global charity fell from $5 in 2005 to $2 in 2020, a 60% decline, due to economies of scale and competition [34].

The favorable experience of residents participating in de-installation suggests that they could easily learn to address minor repairs, such as re-attaching ITWL to a wall if it falls off. If the lifespan could be extended from 4 to 5 years, annualized cost would fall by 19%, i.e., from $10.26 to $8.33 per person. Alternatively, further efficiencies might be achieved by synchronizing de-installation with installation of the next cycle of ITWL in a single day, in the same way that a roofer might remove a damaged roof section and install a replacement at a single visit. In addition, households could be taught safe uses for used ITWL, such as enclosing a latrine, and avoiding its contact with food, thereby reducing the need for removal and incineration of old ITWL.

Overall, we project that efficiencies in installation through stronger management and greater resident involvement, avoiding the need for de-installation and incineration, less expensive ITWL materials, and extending the product’s lifespan could make an ITWL program comparable to or less expensive than IRS.

A feature in Muheza that helped to control cost was the reliance on local, rather than expatriate managers. In Asembo, some personnel came from the U.S. Centers for Disease Control and were paid on international rates. This was a major reason why the overall cost of ITWL per person in Muheza ($39.27) was about half of that in Asembo, $64.23 in 2011 [12], equivalent to $73.69 in 2019 prices. Nevertheless, an economic analysis of the pyrethroid product in Asembo found that even if its lifetime were just 2.2 years, it would be a cost-effective product in areas without pyrethroid resistance [12]. Although one study in India reported a dramatically lower cost per household for ITWL ($8.06) [11] than ours ($157.09), it was not clear whether the Indian study included all the inputs, such as the purchase of the material, full costs of the public health infrastructure, and household time.

Zone B in Fig. 2 shows that if a future ITWL product were more effective than IRS, even at a higher cost, it would still be cost-effective. Several factors could increase the effectiveness of a future ITWL compared to IRS. An installer and a supervisor can both see instantly whether ITWL has been installed correctly on a given wall. On the contrary, as IRS dries quickly, it is difficult for a supervisor to determine whether a wall has been fully sprayed and to confirm that the right dosage has been applied.

High levels of coverage of ITWL would also increase the product’s effectiveness by providing additional protection to neighbors. The final ITWL coverage of 67.7% in Muheza was acceptable, but not excellent. As a community vector control effort, ITWL was intended to protect not only the individual household, but also the community overall. The increases in coverage from phase 1 to 3 were both substantial and relatively rapid, showing that the extra sensitization efforts achieved valuable results. The contrast in the percentage of targeted households covered in Muheza (67.7% for ITWL) and Muleba (93% for IRS) reinforces the importance of community engagement [35]. In Muleba, stakeholders from many levels were engaged in promoting IRS: non-governmental organizations (NGOs) and community-based organizations (CBOs), malaria and health education focal persons, environmental managers, and district and regional officials responsible for IRS [35]. By contrast, the Muheza project involved only a few district officials. Further, their involvement was limited to authorizing the project through issuing letters of introduction, but not actively promoting it.

In summary, several strengths of this study should be noted. Providing a comprehensive measure of resource costs, the study included not only installation costs, but also those of de-installation when the product proved unsuccessful. Second, it disaggregated coverage and costs over three installation phases. Third, it used a societal perspective, including both financial costs within the healthcare system and non-financial costs borne by households.

The research also generated qualitative insights. First, a product more like netting (as installed in Asembo) instead of the sheet-like material (used in Muheza) would be less rigid. This would make installation easier and faster, reduce the risk of the product falling off mud walls, allow air flow if the product is used on the eaves, and lower the temptation for households to re-purpose the product for drying crops. A longer lifespan would also improve the cost-effectiveness. On the other hand, a more effective product could allow a higher cost and still be cost-effective. For example, if ITWL were 100% effective, it could allow a cost per person protected up to $9.98, or only 1.3% below the existing amount, and still be cost effective compared to IRS.

The Muheza experience also highlighted the importance of good communication within a team. In Muheza, all groups’ data were stored in a single system which was supposed to document each day’s progress to plan the next day’s activities. However, the process of retrieving data proved cumbersome, with the data manager needing to attend personally to the demands of various groups to determine where to dispatch teams that day. Sometimes the data became available only after mid-day, resulting in a group losing half or all of a work day. With more detailed planning, the appropriate number of installers could have been hired, trained, and kept busy over all three phases. Changing the payment modality from a daily to a per-house modality lowered the wage bill per household installed by 21%. Thus, more robust data, management, and payment systems would have avoided such bottlenecks and inefficiencies, thereby lowering cost and improving coverage.

If a longer entomological pre-test to measure the product’s efficacy in killing mosquitoes had been possible, the current product’s shortcomings would likely have been identified sooner. The manufacturer might then have been able to modify the product, or the epidemiological study deferred until an entomologically sound product was available.

Finally, while the ITWL installed in Muheza was not effective, data from Asembo and Muheza indicate that a future ITWL has the potential to match or possibly exceed IRS on both usefulness and affordability. Malaria’s worldwide 409,000 deaths in 2019 [5] highlight the need for multiple control measures. Given ITWL’s advantages in implementation, the product merits ongoing investigation starting in the laboratory, and progressing, if results warrant, to experimental huts and African villages.

## Supplementary Information

**Additional file 1.**

## Data Availability

In responding to surveys and providing documents about time, expenses, and payments, household members and staff were promised confidentiality. Inquiries about other unpublished data should be addressed to Dr. Kisinza.

## Abbreviations

CBO: Community-based organization
CHW: Community health worker
DALY: Disability-adjusted life year
GDP: Gross domestic product
GNI: Gross national income
ICER: Incremental cost-effectiveness ratio
IRB: Institutional review board
IRS: Indoor residual spraying
ITWL: Insecticide treated wall liner
LLIN: Long-lasting insecticide treated net
NIMR: National Institute for Medical Research
TRAction: Translating Research to Action
TZS: Tanzanian shilling
USD: United States dollar
