# Supplementary figures and images for: On the cost-effectiveness of insecticide-treated wall liner and indoor residual spraying as additions to insecticide treated bed nets to prevent malaria: findings from cluster randomized trials in Tanzania
Source: BMC Public Health. 2021 Sep 14;21:1666. doi: 10.1186/s12889-021-11671-2 (PMC8439046; doi:10.1186/s12889-021-11671-2)

# CONSORT diagram for Shepard et al.

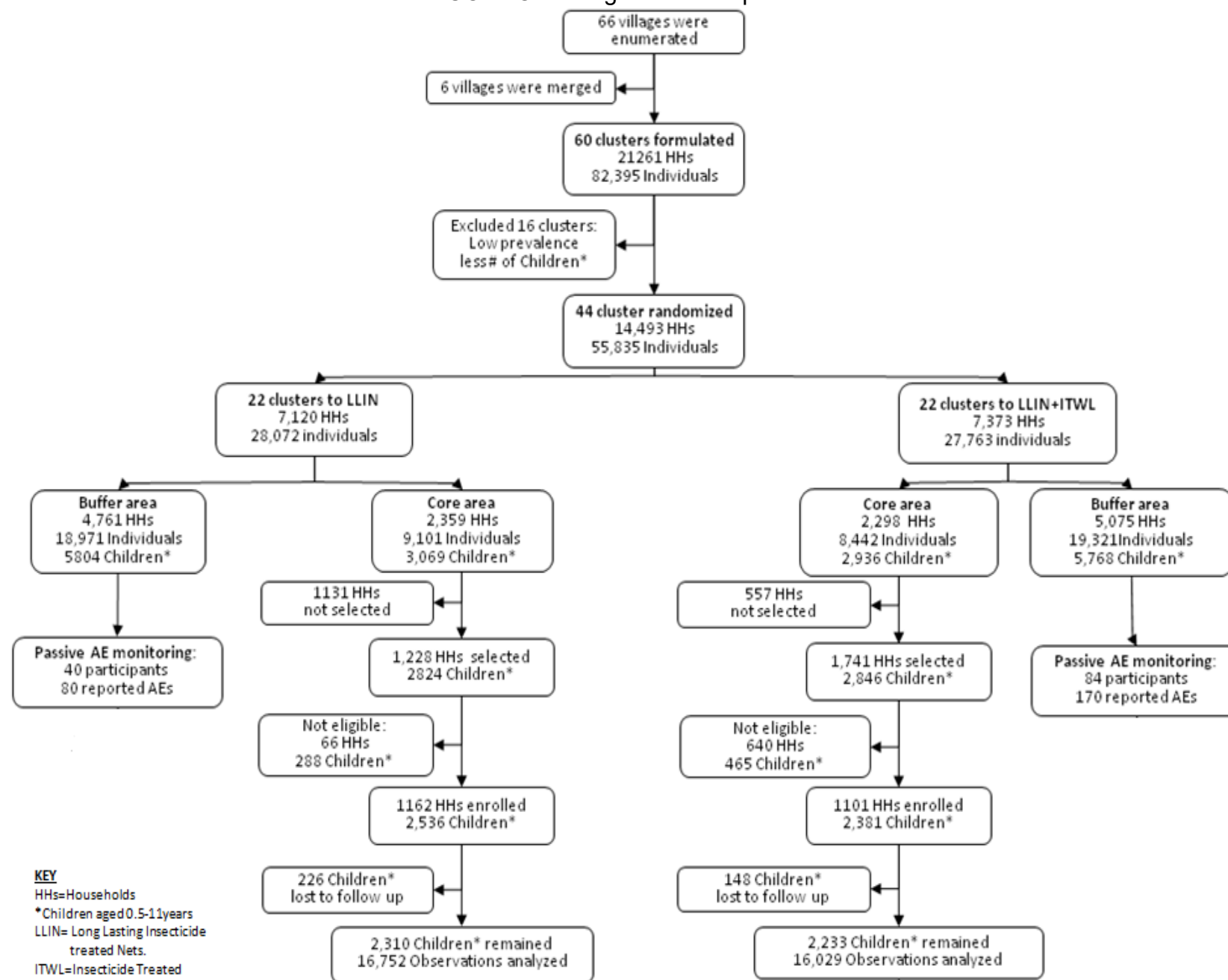

Supplement: Supplementary file 1 — Additional file 1. [file 12889_2021_11671_MOESM1_ESM.pdf]
